# Ecological sensitivity and sustainable spatial planning in a highly urbanized plain: An AHP-GIS and geodetector approach in the Yangtze River Delta Plain

**DOI:** 10.1371/journal.pone.0357598

**Published:** 2026-09-08

**Authors:** Tiantian Yao, Qian Liu, Huiqin Zeng, Jiabin Tang

**Affiliations:** 1 School of Urban Renewal, Shanghai Zhongqiao Vocational and Technical University, Shanghai, China; 2 School of Chemistry and Environmental Engineering, Shanghai Institute of Technology, Shanghai, China; Zhejiang Agriculture and Forestry University: Zhejiang A and F University, CHINA

## Abstract

Rapid urbanization in plains exacerbates ecological pressures, while most ecological sensitivity (ES) research focuses on mountains, offering limited guidance for highly urbanized plains. This study assesses ES in the Yangtze River Delta Ecological Green Integrated Development Demonstration Zone (YRD EGI-DDZ) and its planning implications. Ten indicators, covering four criteria, geography, hydrology, natural resource, and human interference were integrated using Analytic Hierarchy Process (AHP) with Geographic Information System (GIS). Geodetector quantified drivers of ES spatial variation, and 2018–2020 land use data revealed recent development and fragmentation. Results show over 70% of the area under medium to extremely highly sensitivity, with high or extreme high zones clustered along Dianshan Lake, Yuandang, East Taihu, and contiguous ecological land. Low sensitivity mainly corresponds to urban land. Key drivers include land use, nighttime lights, population density, and water proximity, while elevation and slope contribute marginally. New construction land expands mainly along urban fringes and transport corridors, inserting low sensitivity strips into medium sensitivity belts and fragmenting high sensitivity cores. These findings inform a sensitivity-based zoning and corridor control framework, refining existing plans and supporting sustainable spatial development in similar plains.

## 1. Introduction

Rapid urbanization and industrialization have substantially intensified ecological and environmental pressures in many regions worldwide. This conflict is particularly acute in large, flat riverine metropolitan regions, which are often economic powerhouses characterized by high population density, complex hydrological networks, and fragmented governance across administrative boundaries [1]. The Yangtze River Delta in this study specifically refers to the Yangtze River Delta Ecological and Green Integrated Development Demonstration Zone (YRD EGI-DDZ), a national strategic practice zone approved by State Council in 2019. It is also a typical example of such a highly urbanized plain river and lake system, where dense water networks, intensive land use change, and cross jurisdictional management pose significant challenges for environmental sustainability [2,3]. Against this backdrop, ecological sensitivity (ES) assessment has emerged as an important tool for identifying areas that are more susceptible to disturbance and for supporting ecological protection zoning, land use regulation, and sustainable spatial planning.

ES describes how strongly an ecosystem reacts to external stressors such as soil erosion, sandstorms, extreme snowfall events, or human disturbance. It reflects both the susceptibility of ecosystem structure and processes to environmental change and the system’s ability to recover and adapt [4]. In practice, ES is typically assessed by integrating multiple natural and anthropogenic factors and mapping their spatial distribution. Such assessments provide a scientific basis for prioritizing conservation, delineating development boundaries, and designing differentiated management strategies [5–7]. As a key concept in environmental management and conservation, ES assessment provides a scientific basis for prioritizing conservation, delineating development boundaries, identifying protection hotspots, and mitigating ecological risks. Consequently, once ES has been mapped and classified, its results are often further interpreted and translated into conventional land use suitability evaluations to better inform spatial planning and policy decisions [8,9].

Due to ES assessment inherently involves multiple, heterogeneous indicators and requires their integration in a spatially explicit way, it naturally lends itself to multi‑criteria decision‑making and GIS‑based analysis frameworks. In this context, combining a transparent weighting method with spatial overlay tools has become a common and effective practice. The integration of the AHP with GIS is a well-established technique in Multi-Criteria Decision-Making (MCDM-GIS). This approach provides a structured mechanism for weighting and combining various spatial criteria [10]. Leveraging GIS capabilities, it effectively synthesizes disparate data sources to support rapid and robust spatial evaluation [11]. Consequently, owing to these strengths in systematic factor integration and visualization, AHP-GIS has been widely adopted for ES assessment or space suitability assessment, particularly in fragile regions, as demonstrated in numerous studies [12–16]. Nevertheless, a key limitation of AHP lies in the subjectivity of the expert-based weight assignment, and maintaining consistency becomes increasingly difficult as the number of criteria grows. To address the inherent limitations of discrete pairwise comparisons in conventional AHP, several studies have adopted Fuzzy AHP (FAHP) or FAHP-CV, FAHP, AHP and F-TOPSIS to assess weight perturbations [17–20]. These approaches aim to reduce subjectivity and enhance robustness. However, a fundamental issue remains: indicator weights in AHP-GIS are still essentially based on expert scoring and thus cannot avoid subjectivity, and most applications are static, evaluating sensitivity only under current conditions without an independent, data-driven check on the assumed factor importance. To address these shortcomings, this study couples AHP-GIS with Geodetector and land use change (LUC) analysis. AHP-GIS is used to construct the ES model and derive initial weights, while Geodetector quantifies the explanatory power of each factor for the spatial differentiation of ES, providing an objective basis to examine and interpret the AHP derived weights and to better align the weighting scheme with the empirical spatial pattern. In addition, LUC analysis between 2018–2020 is used to relate recent land use dynamics to ES fragmentation, thereby enhancing the interpretability and planning relevance of the sensitivity mapping.

Meanwhile, there are also some limitations for the ES study of YRD EGI-DDZ. First, most existing ES studies have concentrated on regions with pronounced relief mountains and plateaus such as the Qinghai Tibet Plateau [21], the Loess Plateau, and Southwest China, where geographical factors are dominant controls and AHP-GIS multi-factor overlays have been widely applied. By contrast, work on low-lying plains with dense river networks and intense urbanization, such as the Yangtze River Delta plain, is relatively scarce, and many methods and indicator systems are essentially “mountain-oriented” transplants that have not been systematically adapted to plain-dominated mechanisms. In particular, the shift of ES drivers in low-relief plains, from geographical control toward hydrological processes, land use structure, and human disturbance, that remains insufficiently understood, leaving a clear gap in comprehensive ES assessment frameworks explicitly tailored to plain regions. Second, existing researches on the YRD EGI-DDZ and the broader Yangtze River Delta mainly focus on ecosystem services supply and demand relationship and trade-off of ecosystem services, or use InVEST and related models to analyze habitat quality dynamics [22–25], as well as the construction of ecological networks, security patterns and spatial synergy [26, 27]. These works are largely service or network oriented, and although they consider natural and socio-economic drivers (e.g., climate, land use, population, nighttime lights), they do not provide a comprehensive, plain-specific ES framework that systematically integrates multiple natural and human disturbance factors. However, these studies also do not combine expert-based MCDM weighting with data-driven factor detection to identify dominant drivers in a low-relief, highly urbanized river and lake system. Nevertheless, most existing work remains centered on single dimension ecological indicators (e.g., water purification) and retrospective analyses of past changes, while comprehensive ES frameworks that explicitly integrate geography, hydrology, natural resources, and multi-source human disturbances in a low relief setting are still limited. Moreover, most analyses are rarely translating results into sensitivity-oriented land suitability and zoning tools.

In summary, several critical research gaps persist in the current studies. First, at the methodological level, AHP-GIS applications still rely heavily on expert judgement, and the resulting weights lack independent, data driven validation. Second, in terms of research content, the frameworks for ES assessment are still predominantly oriented towards mountainous and plateau regions. Their indicator systems and weighting schemes have rarely been systematically recalibrated for low-relief plains and are often directly transplanted, leading to potential mismatches with plain dominated mechanisms. Third, within the YRD EGI-DDZ and the broader Yangtze River Delta, many studies remain service or network oriented, focus on single dimension ecological metrics and retrospective analyses (e.g., 2000–2020 habitat quality or ecosystem services), and lack a comprehensive, plain specific ES framework.

Therefore, against this background, this paper makes three key contributions: (1) It develops an AHP-GIS ES framework specifically tailored to the low-lying, highly urbanized YRD EGI-DDZ, integrating geographical sensitivity, hydrological sensitivity, natural ecological sensitivity (soil, land use, vegetation) and human interference sensitivity (roads, population, nighttime light) factors, and elucidating a shift in dominant drivers from geographical control toward land use structure, human activities and hydrological proximity in plain areas. (2) It combines expert based AHP weighting with Geodetector to quantify each factor’s explanatory power for the spatial differentiation of ES, providing data driven validation and interpretation of the AHP derived weights and enabling a more robust identification of dominant drivers in the plain context. (3) It innovatively links ES assessment with recent LUC analysis (2018–2020), showing how expansion along urban fringes and transport corridors wedges into medium sensitivity belts and fragments high sensitivity cores, and on this basis translates ES maps into sensitivity-oriented protection regulation development zoning and corridor control suggestions, offering direct support for territorial spatial planning in the YRD EGI-DDZ.

## 2. Materials and methods

### 2.1. Study area

**Fig 1:**
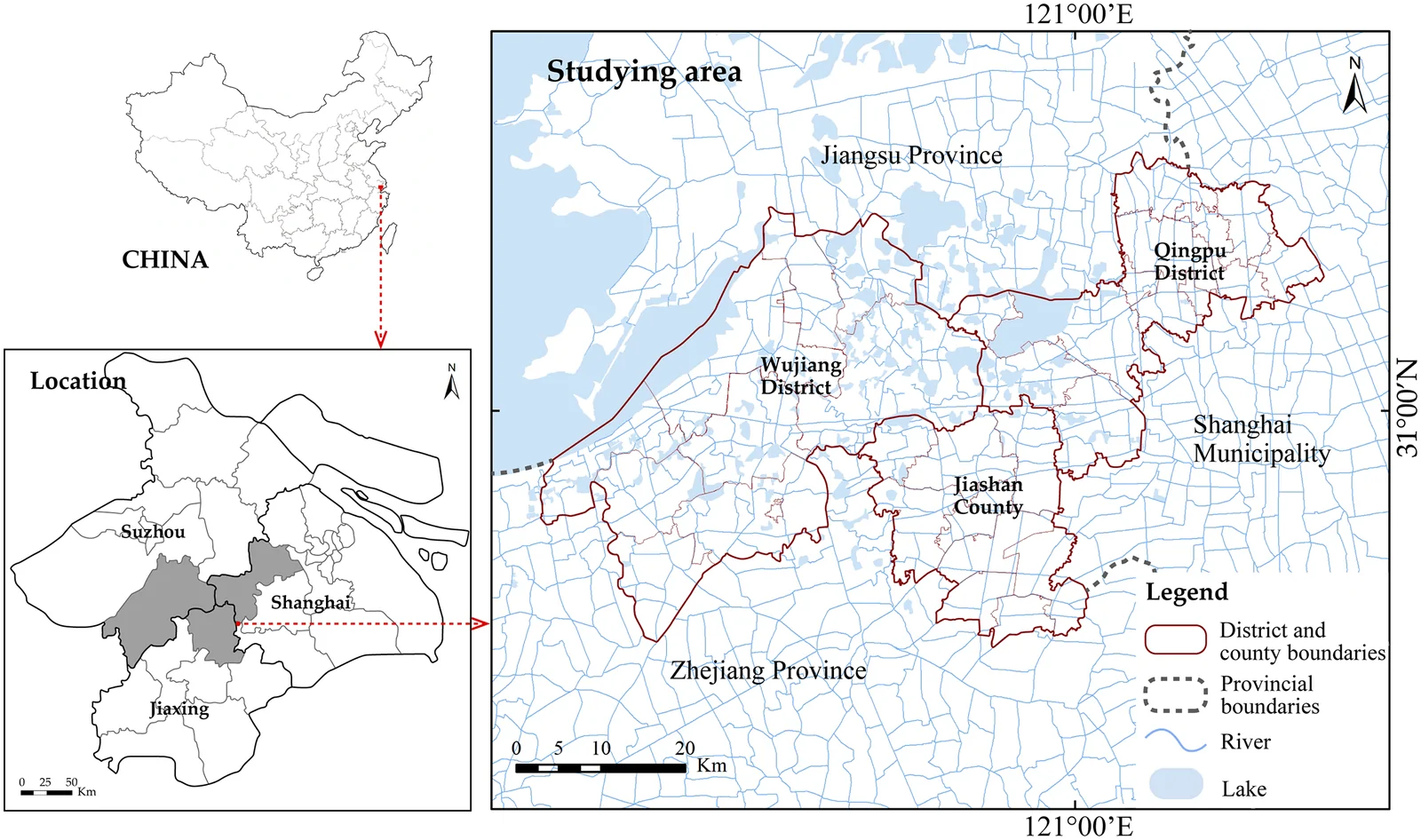
Location. Base map data obtained from the China Standard Map Service, Ministry of Natural Resources of the People’s Republic of China (http://bzdt.ch.mnr.gov.cn/). Map approval number: GS (2020) 3189.

This study focuses on the YRD EGI-DDZ, a core area within the Yangtze River Delta urban agglomeration in eastern China (Fig 1). Located at the junction of Shanghai, Jiangsu, and Zhejiang provinces, covering Qingpu District of Shanghai, Wujiang District of Suzhou City in Jiangsu Province and Jiashan County of Jiaxing City in Zhejiang Province (E120°–121°, N30°–31°). With a total area of approximately 2,413 km², the demonstration zone is characterized by a dense network of rivers and lakes, flat geography, and a high level of urbanization, situated within a subtropical monsoon climate zone. As one of China’s most dynamic economic cores, the YRD EGI-DDZ faces significant pressures from rapid urban expansion, infrastructure development, and intensive agriculture, leading to habitat loss, landscape fragmentation, non-point source pollution, and increased flood risks [28, 29]. Concurrently, the region bears the strategic mandate of exploring pathways for ecological and green integrated development and cross-jurisdictional ecological collaborative conservation. Against this backdrop, there is a critical need for the YRD EGI-DDZ to establish a scientifically sound ES assessment framework to inform decision-making for implementing spatial strategies that prioritize ecology development.

### 2.2. Data sources and research framework

The ES assessment framework of this study is based on multiple datasets spanning geography, hydrology, natural resource, and human interference (Table 1). It should be explained that human interference was defined as the intensity of human activities exerting pressure on ecosystems. It was operationalized through three GIS-based indicators: nighttime lights, population density, and road buffer zones. These indicators were selected based on their established relevance to ecological disturbance and were integrated into the AHP weighting framework. These data were uniformly imported into ArcGIS 10.8 for preprocessing to derive multi-factor information for the study area, including elevation, slope, and water body buffers etc. All data were standardized to the WGS_1984_UTM_Zone_51N coordinate system and resampled to a unified resolution of 30-meter×30-meter to ensure analytical consistency and compatibility. To be specific, the road data encompass both highways and railways; Fractional Vegetation Cover (FVC) can be measured via remote sensing indices such as the Normalized Difference Vegetation Index (NDVI), which is the most widely used indicator in ecological research [30, 31]. For this study, the NDVI data were derived from the Google Earth Engine (GEE) cloud-computing platform. Annual maximum NDVI values were calculated using Landsat 8 imagery, specifically based on bands B5 and B4. The nighttime light data originated from the Visible Infrared Imaging Radiometer Suite (VIIRS) onboard the Suomi National Polar-orbiting Partnership (SNPP) satellite, processed to generate annual nighttime light brightness datasets. The research data access date for all datasets was January 10, 2026.

**Table 1:** Data source.

Name of Data	Spatial resolution	Period	Product Source	Data sources
DEM	30m	2021	GDEMV 3	https://www.gscloud.cn/ Geospatial Data Cloud
River	–	2022	–	https://www.tianditu.gov.cn/ Mapworld
Lake				
Surface type	30m	2025	–	https://gis5g.com/ Earth Resources Data Cloud
Land use	30m	2020	Landsat 8	https://www.resdc.cn/ Resource and Environmental Science Data Platform
NDVI	30m	2021		
Road	–	2022	–	https://www.tianditu.gov.cn/ Mapworld
Population	30m	2024	LandScan HD	https://landscan.ornl.gov/ OAK RIDGE National Laboratory
Nighttime light data	500m	2021	NPP- VIIRS	https://www.resdc.cn/ Resource and Environmental Science Data Platform

All selected data are the latest and publicly available.

**Fig 2:**
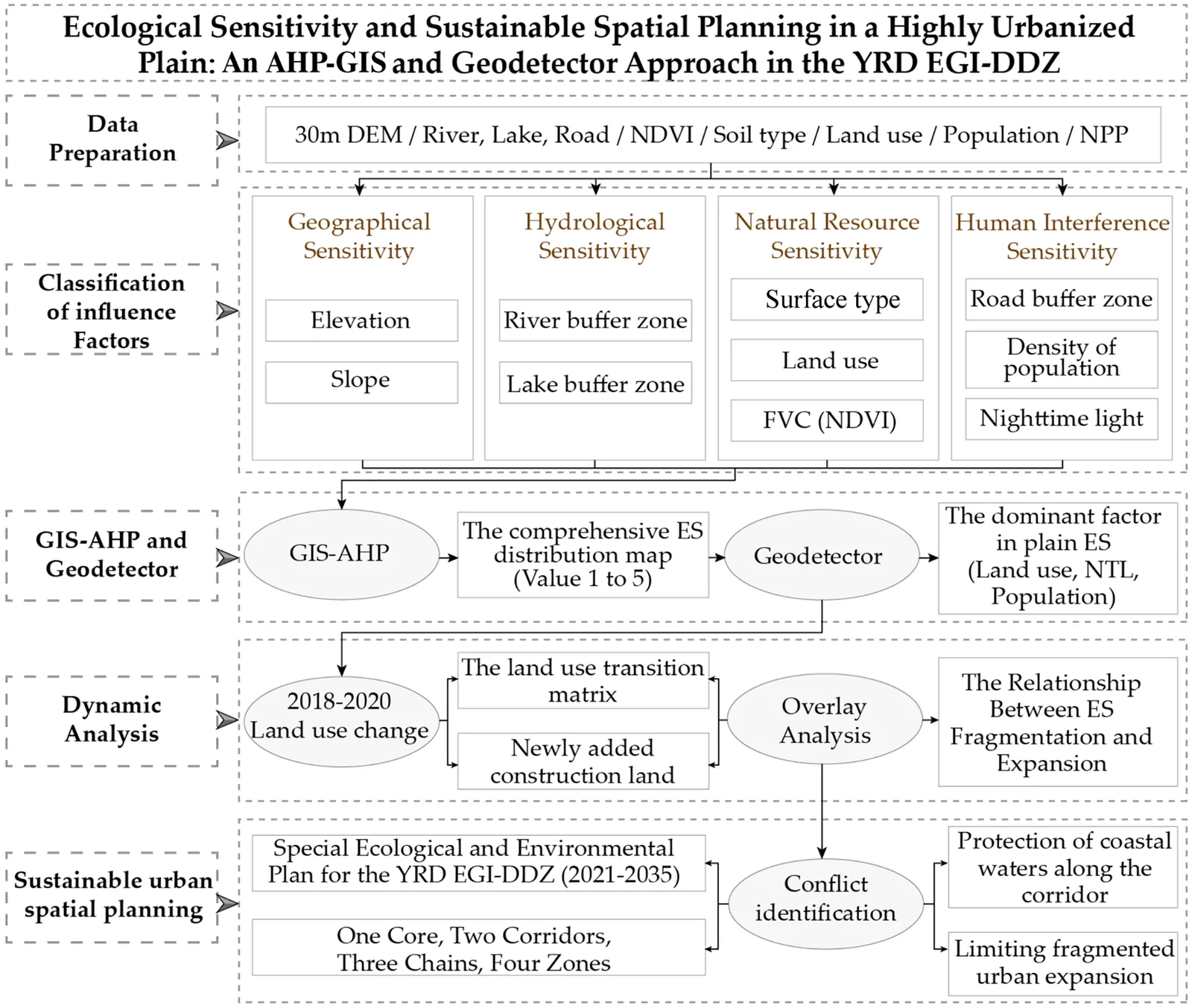
The research framework.

The research framework consists of four main steps (Fig 2). First, ten indicators describing geography, hydrology and ecological base, and human disturbance are organized into a four level ES index system and weighted using AHP in a GIS environment to produce a comprehensive ES map. Second, Geodetector is applied with ES classes as the dependent variable and the classified indicators as explanatory variables to identify dominant drivers and to verify and interpret the AHP derived weights in a highly urbanized plain context. Third, land use data for 2018 and 2020 are used to build a land use transition matrix, identify new construction expansion along urban fringes and transport corridors, and relate these changes to ES fragmentation. Finally, through a comprehensive analysis of the ES model, dominant driving factors, and land use dynamics, we propose sensitivity-oriented recommendations for protection, regulation, development zoning, and corridor control, providing a basis for sustainable spatial planning in the demonstration area.

### 2.3. Modelling Ecological Sensitivity (ES)

Ecosystem complexity dictates that the selection of ES factors cannot be standardized but is contingent upon specific environmental contexts [32–34]. Accordingly, this study proposes a comprehensive multi-factor assessment framework. In contrast to prior studies focused on plateau and mountainous regions, the present framework is specifically tailored to the unique characteristics of the study area. It prioritizes hydrological, natural, and human Interference factors, while assigning a comparatively lesser weight to purely geographical determinants. Derived from an extensive synthesis of prior research, each dimension further subdivided into specific influencing factors (Table 2). Each normalized evaluation factor was categorized into five distinct sensitivity classes: insensitivity, slightly sensitivity, moderately sensitivity, highly sensitivity, and extremely sensitivity. At the same time, using the Natural Breaks Method (Jenks Natural Breaks) on the basis of data distribution (Elevation, Slope, Density of population and Nighttime light). However, since surface types cannot be numerically classified, the surface types were reclassified into five sensitivity levels (1–5) based on a composite criterion that considers: (1) the degree of naturalness of the soil ecosystem, (2) its coupling intensity with hydrological and wetland processes, and (3) the potential impact of anthropogenic development on its ecological functions. Accordingly, soils with high naturalness, strong hydrological connectivity, and high vulnerability to disturbance were assigned higher sensitivity scores (e.g., lakes and water bodies), whereas those with lower naturalness and weaker ecological functions received lower scores. Corresponding importance scores of 1–5 were assigned to these classes. In the GIS environment, the factor raster was subsequently reclassified into sensitivity maps with these ordinal values (1–5) via the Reclassify function, establishing a unified single-factor grading criterion for ES in the YRD EGI-DDZ.

**Table 2:** Grading criteria of ES evaluation factors.

Objective Layer	Criterion Layer	Indicator Layer	Natural Breaks Method				
Ecological Sensitivity	Geographical Sensitivity	Elevation/m	<9	9-13	13-21	21-43	>43
		Slope/°	<1	1-3	3-6	6-13	>13
	Hydrological Sensitivity	River buffer zone/m	>800	600-800	400-600	200-400	<200
		Lake buffer zone/km	>20	15-20	8-15	3-8	<3
	Natural Resource Sensitivity	Surface type	Take off the latent paddy soil	Paddy Soils	Gleyed paddy soil & Fluvoaquic Soils	Fluvial Sand Deposits& Waterloggogenic paddy soil	Lakes and water bodies
		Land use	Urban and Construction land	Rural and Dry land	Paddy fields & Sparse/ other woodland	Forest land & High coverage grassland& Tidal flats & Floodplains	Marshland & Reservoirs and ponds & Rivers and Lakes & Canals
		FVC (NDVI)	<0.2	0.2-0.4	0.4-0.6	0.6-0.8	>0.8
	Human Interference Sensitivity	Road buffer zone/m	<100	100-200	200-500	500-1000	>1000
		Density of population	>514	197-514	58-197	18-58	< 18
		Nighttime light	> 41	26-41	15-26	1-15	<7
Grading			1	2	3	4	5
Degree			In- sensitivity	Slightly sensitivity	Moderately sensitivity	Highly sensitivity	Extremely sensitivity

In summary, grounded in the existing literature and considering data authenticity and availability, this research constructed an ES assessment system tailored to the characteristics of the study area. The system integrates ten key indicators: elevation, slope, river buffer zone, lake buffer zone, surface type, fractional vegetation cover (FVC), land use, road buffer zone, population density, and nighttime light. The rationale for selecting these indicators is detailed below (Table 3)

**Table 3:** Indicator selection principle.

Criterion Layer	Indicator Layer	Selection Principle
Geographical Sensitivity	Elevation	Elevation directly determines ecosystem vulnerability, with plains being more susceptible to human disturbance compared to high-altitude areas [35].
	Slope	Governs runoff and erosion; The steeper slopes are more sensitive to change.
Hydrological Sensitivity	River buffer zone	Critical, sensitive ecotones for water quality, habitat, and flood regulation. Areas near water bodies typically exhibit greater ES [36]. The YRD EGI-DDZ features a dense waterway network, where buffer zone designation aids in identifying and safeguarding ecologically sensitive areas.
	Lake buffer zone	Core areas for water and habitat; It is sensitive to pollution and physical alteration. The closer the distance, the higher the ES.
Natural Resource Sensitivity	Surface type	Determines fundamental hydrologic and stability traits.
	Land use	All human socio-economic activities are based on land. Therefore, as a key interface between human systems and the natural environment, land use underpins most anthropogenic environmental problems [37–39]. In particular, the highly urbanized area like YRD EGI-DDZ is experiencing increasing pressure on land resources, leading to ecological and environmental problems, which is of great significance for studying ES.
	FVC (NDVI)	FVC is an important indicator for quantifying vegetation density and reflecting vegetation growth, while also acting as a fundamental proxy for ecological environment dynamics [40]. Rapid urban expansion often triggers vegetation loss and degradation, thereby impacting ES.
Human Interference Sensitivity	Road buffer zone	Linear sources of habitat fragmentation and concentrated disturbance. The farther away from the road, the better the ES.
	Density of population	Population density serves as a key proxy for human activity intensity and ecosystem pressure, and is typically negatively correlated with ES.
	Nighttime light	The data were used as a proxy for human disturbance intensity. Since higher NTL values indicate more intensive human activities, the relationship between NTL and sensitivity was considered negative.

### 2.4. Weight assignment

To derive the indicator weights for the AHP‑based ES assessment, we convened an expert panel following a structured selection procedure. Eight experts were invited, all holding doctoral degrees in relevant fields (e.g., landscape architecture, urban and regional planning, environmental science, and ecology), with peer-reviewed publications in spatial planning, ecological assessment, or direct experience in the Yangtze River Delta region. The panel was deliberately composed to represent diverse institutional perspectives—five experts from universities, two from research institutes, and one from a public planning agency, thereby integrating theoretical, empirical, and practice‑oriented knowledge into the weighting process.

Data were collected through online and in-person interviews, depending on each expert’s availability. The expert survey, entitled “Assessment of ES Factors in the YRD EGI-DDZ,” employed pairwise comparisons based on the Saaty 1–9 scale (Table 4). Experts were asked questions such as: “For the ecological environment of the demonstration zone, which do you consider more important, hydrology or geography, and to what extent?” Within each hierarchical level, comprising four first-level criteria and ten second-level indicators. Factors were compared in pairs, and each expert’s judgments were used to construct a corresponding judgment matrix A.

**Table 4:** Saaty 1–9 scale.

Scale	Interpretation
1	The contributions of two variables are equally important
3	One variable is slightly important than other
5	One variable is significantly important than other
7	One variable is very strong important than other
9	One variable is extremely important than other
2,4,6,8	Intermediate important
Reciprocal Property	If the importance ratio of row factor i to column factor j is denoted as $a_{ij}$, then the importance ratio of factor j to factor i is $a_{ji}$ =$\text{1}/a_{ij}$


$$\begin{array}{c} Judgment\;matrix\;A\;=\;\;\left\{ \begin{array}{cccc} \text{1} & a_{\text{1}\text{2}} & \cdots & a_{\text{1}n}\\ \frac{\text{1}}{a_{\text{1}\text{2}}} & \text{1} & \cdots & a_{\text{2}n}\\ \cdots & \cdots & \ddots & \cdots \\ \frac{\text{1}}{a_{\text{1}n}} & \frac{\text{1}}{a_{\text{2}n}} & \cdots & \text{1} \end{array}\right.\;\} \end{array}$$ (1)

The element ${\text{a}}_{\text{ij}}$ represents the relative importance ratio of the i-th element compared to the j-th element. It satisfies the conditions that ${\text{a}}_{\text{ij}}$ > 0, ${\text{a}}_{\text{ij}}$ = $\frac{\text{1}}{{\text{a}}_{\text{ji}}\;}$, and ${\text{a}}_{\text{ii}}$ = 1.

With multiple experts participating, the geometric mean of each row in judgment matrix A was calculated:


$$\begin{array}{c} G_{i}\;=\;\sqrt[{n}]{\prod_{j=\text{1}}^{n}aij},\;(i=\text{1},\text{2},\;\text{3}...,\;n) \end{array}$$ (2)

The vector G = T was then normalized to obtain the weight vector W = T:


$$\begin{array}{c} W_{i}\;=\frac{Gi}{\sum_{j=\text{1}}^{n}Gj} \end{array}$$ (3)

Where ${\text{W}}_{\text{i}}$ denotes the weight of the corresponding factor. Subsequently, a consistency check was conducted by computing the maximum eigenvalue $\lambda_{\text{max}}$:


$$\begin{array}{c} \lambda_{max}=\;\sum_{i=\text{1}}^{n}\frac{(AW)i}{nWi} \end{array}$$ (4)

Where (AW)i represents the i-th element of the vector AW. This was followed by the calculation of the consistency index (CI):


$$\begin{array}{c} CI=\;\frac{\lambda_{max}\;-\;n}{n-\text{1}} \end{array}$$ (5)

Using the random consistency index (RI) obtained from standard tables, the consistency ratio (CR) was then calculated:


$$\begin{array}{c} CR=\;\frac{CI}{RI} \end{array}$$ (6)

CR < 0.10 indicates acceptable matrix consistency.

After consistency checking, individual matrices were aggregated and normalized to obtain the final AHP weights used in the GIS based ES modelling.

### 2.5. Integration of weighted fctors for comprehensive ES evaluation

The comprehensive ES of the YRD EGI-DDZ was evaluated through a GIS based weighted overlay analysis. This was applied by combining the reclassified sensitivity maps of each individual factor with their corresponding AHP derived weights. The specific assessment model is formulated as follows:


$$\begin{array}{c} S_{j}\;=\sum_{i=\text{1}}^{n}WiFi \end{array}$$ (7)

Where ${\text{S}}_{\text{j}}\;$represents the comprehensive score of ES, Wi denotes the weight of the i-th indicator, Fi refers to the score of the i-th indicator, N is the total number of indicators.

### 2.6. Geodetector model

To identify the dominant drivers of ES in the study area, Geodetector was applied using the ES class as the dependent variable and the classified indicators as explanatory factors. To reduce redundancy while maintaining spatial representativeness, a total of 5,000 grid cells were randomly sampled from the full set of more than 2.8 million cells, and for each sample the ES class (Y) and all factor classes (X) were extracted. The factor detector module was then used to compute the q-statistic and associated p value for each indicator.

To analyze the spatial heterogeeity of factors affecting ES, this study employed the Geodetector model. This statistical tool, developed by Wang, typically operates through four detectors (factor, interaction, risk, and ecological) [41]. This classification provides a straightforward reference for comparing the relative importance of different drivers in shaping ES spatial patterns.We specifically utilized the factor and interaction detectors. The explanatory strength of the influencing factors was measured by the factor detector’s q-statistic, with the corresponding equation presented below:


$$\begin{array}{c} q\;=\;\text{1}-\;\frac{\sum_{k=\text{1}}^{M}N_{k}\sigma_{k}^{\text{2}}}{N\sigma^{\text{2}}} \end{array}$$ (8)

The q-statistic, ranging from 0 to1, measures the explanatory power of each factor on ES spatial distribution. A higher q-value indicates that a greater proportion of the spatial variance of ES is explained by the corresponding factor. This metric serves as a direct measure of some factor’s relative importance in shaping the observed ES pattern. Furthermore, following the established practice in Geodetector-based studies, the explanatory power of each factor was classified into three tiers based on the q-statistic value: strong (q ≥ 0.30), moderate (0.10 ≤ q < 0.30), and weak (q < 0.10). Where k represents the number of strata for the factor; M is the number of categories; ${\text{N}}_{\text{k}}$ and N represents the number of samples in layer k and the whole region; and $\sigma_{\text{k}}^{\text{2}}$ and $\sigma^{\text{2}}$ are the variances in layer the value of q.

To assess factor interactions, we applied the interaction detector. This method evaluates whether the combined effect of two factors on ES is enhanced or weakened compared to their individual effects, following the criteria summarized in Table 5.

**Table 5:** The basis for judging two-factor interaction patterns.

Criterion	Interaction Type
q(${\text{X}}_{\text{1}}\cap {\text{X}}_{\text{2}}$) <Min (q(${\text{X}}_{\text{1}}),\text{ q}({\text{X}}_{\text{2}})$)	Nonlinear weakening
Min (q(${\text{X}}_{\text{1}}),\text{q}({\text{X}}_{\text{2}})$) <q(${\text{X}}_{\text{1}}\cap {\text{X}}_{\text{2}}$) <Max (q(${\text{X}}_{\text{1}}),\text{ q}({\text{X}}_{\text{2}})$)	Single nonlinear enhancement
q(${\text{X}}_{\text{1}}\cap {\text{X}}_{\text{2}}$) > Max (q(${\text{X}}_{\text{1}}),\text{ q}({\text{X}}_{\text{2}})$)	Double enhancement
q(${\text{X}}_{\text{1}}\cap {\text{X}}_{\text{2}}$) = q(${\text{X}}_{\text{1}})+\text{q}({\text{X}}_{\text{2}})$	Independence
q(${\text{X}}_{\text{1}}\cap {\text{X}}_{\text{2}}$) > q(${\text{X}}_{\text{1}})+\text{q}({\text{X}}_{\text{2}})$	Nonlinear enhancement

### 2.7. Land use change (LUC) from 2018 to 2020

To explore how recent land use dynamics have shaped the current ES pattern, land use between 2018 and 2020 was analyzed using the multi-temporal datasets. However, the land use analysis is purely descriptive and based on cross-tabulation between the 2018 and 2020 land use maps, no Markov-chain prediction was applied.

For this analysis, the original land use categories were reclassified into six functional types (1–6): cropland, woodland, grassland, water bodies, construction land, and unused land. A transition matrix for 2018–2020 was then generated using GIS. This matrix was used to quantify the main conversion processes (e.g., cropland to construction land, ecological land to construction land) and the relative stability of each category. Then, to link LUC with sensitivity, the 2018 land use map was reclassified into five sensitivity levels (1–5) according to ecological characteristics, consistent with the 2020 map. For each year, the area and proportion of land in each sensitivity level were calculated and compared to reveal structural shifts in land-use-related sensitivity between 2018 and 2020, providing a basis for interpreting the observed high and fragmented ES pattern in the YRD EGI-DDZ.

## 3. Results

### 3.1. ES analysis of single factor

**Fig 3:**
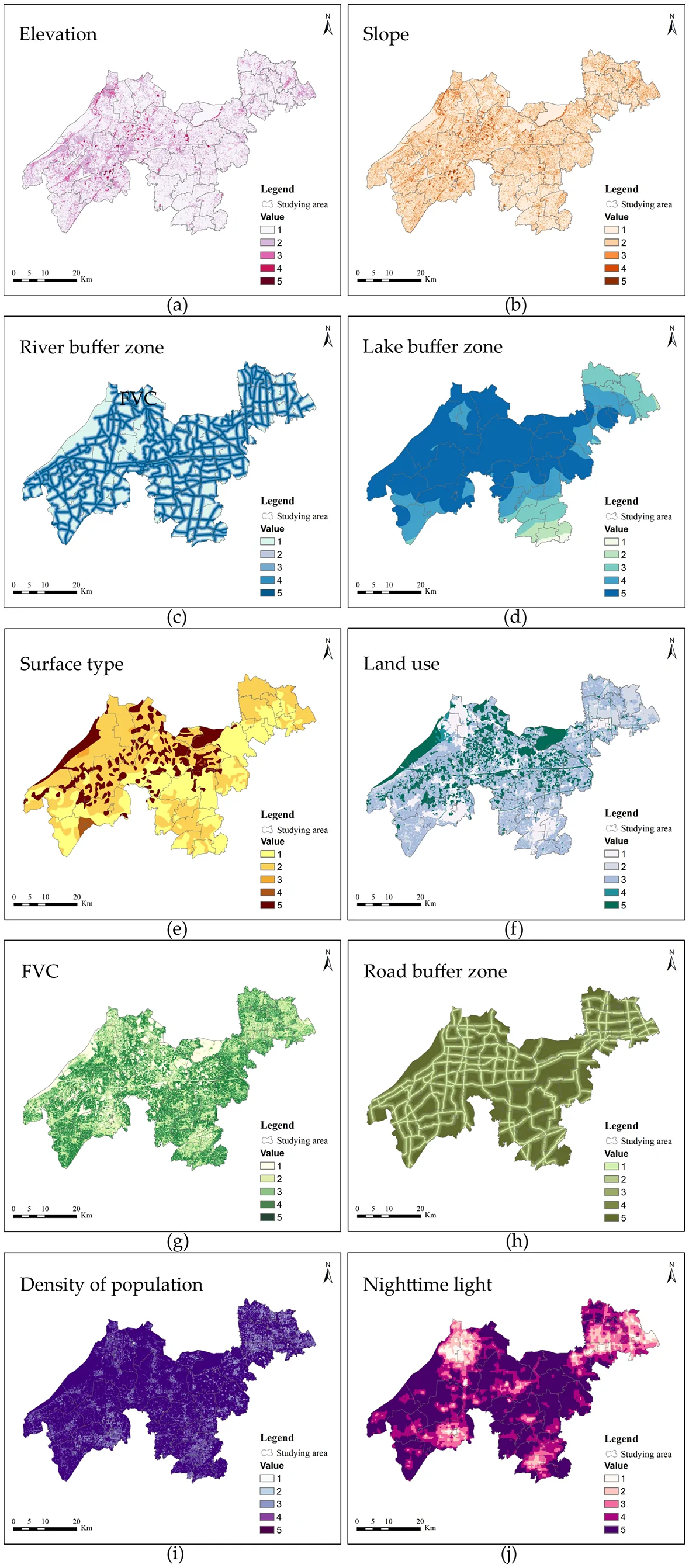
Distribution maps of ES. a. Elevation; b Slope; c. River buffer zone; d. Lake buffer zone; e. Surface type; f. Land use; g. FVC; h. Road buffer zone; i. Density of population; j. Nighttime light. Base map data obtained from the China Standard Map Service, Ministry of Natural Resources of the People’s Republic of China (http://bzdt.ch.mnr.gov.cn/). Map approval number: GS (2020) 3189.

Through GIS data processing, the distribution maps of ES for various factors of YRD EGI-DDZ were obtained (Fig 3), along with the areas of each factor at different sensitivity levels (Table 6). Specifically, the demonstration zone covers an area of about 241300 ha (2,413 km²) as officially reported. Based on the GIS boundary used in this study, the calculated area is 240343.4 ha (2,403.43 km²), which is within an acceptable margin of error.

**Table 6:** Proportion of sensitivity area for various evaluation grades (based on multi-source data from 2020-2025). land use (2020), DEM, NDVI and nighttime lights (2021), River and lake, and road (2022), population (2024), and surface type (2025).

Evaluation index system		Proportion of sensitivity area for various evaluation grades				
Criterion Layer	Indicator Layer	In- sensitivity	Slightly sensitivity	Moderately sensitivity	Highly sensitivity	Extremely sensitivity
Geographical Sensitivity	Elevation	69.01%	24.75%	5.43%	0.75%	0.06%
	Slope	52.25%	31.34%	13.32%	2.79%	0.30%
Hydrological Sensitivity	River buffer zone	24.16%	12.67%	16.88%	21.35%	24.94%
	Lake buffer zone	0.66%	3.02%	12.31%	19.73%	64.28%
Natural Resource Sensitivity	Surface type	35.26%	47.22%	0.45%	0.85%	16.22%
	Land use	14.69%	16.17%	47.42%	0.97%	20.75%
	FVC	14.77%	25.64%	26.15%	33.06%	0.38%
Human Interference Sensitivity	Road buffer zone	9.35%	8.76%	22.77%	26.39%	32.73%
	Density of population	0.02%	0.09%	6.20%	18.33%	75.36%
	Nighttime light	2.53%	6.54%	11.29%	22.65%	56.99%

First, the geographical factors exhibited the lowest overall sensitivity. The distribution of sensitivity levels for the evaluation indicators Elevation (Fig 3a) and Slope (Fig 3b) shows a high degree of consistency, with high-value areas being scattered and limited in extent. In the sensitivity level statistics, the combined area of insensitivity and slightly sensitivity far exceeds that of moderately and highly sensitivity, with only minimal areas classified as highly and extremely sensitivity. This pattern aligns with the overall flat terrain and extremely low spatial variability in elevation and slope across the YRD EGI-DDZ. It indicates that elevation and slope contribute very little to the spatial differentiation of ecological sensitivity in this region, functioning more as background factors.

In contrast, the hydrological proximity factors display significant belt-like high sensitivity features. Both the River buffer zone (Fig 3c) and Lake buffer zone (Fig 3d) reveal continuous belts of highly and extremely sensitivity along the banks of major rivers and tributaries (e.g., Taipu River, Beijing-Hangzhou Grand Canal) and lakes (e.g., East Taihu Lake, Dianshan Lake, Yuandang Lake). Furthermore, for both River and Lake factors, the area classified as highly sensitivity and above accounts for nearly or over 50% of the total ecologically sensitive area. Particularly notable are the extensive, contiguous extremely sensitivity areas around lakes, constituting 64.28% of the lake buffer zone. This reflects that in low-relief plain river network areas, water body shorelines and land-water transition zones are critical for maintaining regional hydrological processes and habitat connectivity, making them extremely sensitive to occupation and hardening.

Among the natural factors, land use and FVC exert the strongest discriminatory effect. Most areas for the regional surface type (Fig 3e) are classified at moderately sensitivity or below, with areas above this level totaling only 17.52%. This suggests that different soil combinations have a moderate influence on hydrology, fertility, and ecological processes. Conversely, land use (Fig 3f) and FVC (Fig 3g) show that highly and extremely sensitivity areas primarily correspond to forestland, high-coverage grassland, wetlands, and some non-constructed farmland. Construction land and some dry farmlands are concentrated in the slightly sensitivity and insensitivity levels. Overall, the structure of land use and FVC determines the spatial pattern of “ecological baseline quality” in this region and represents the most significant contributing factors among the natural categories.

The human disturbance factors exhibit a typical pattern of “extremely sensitivity Versus insensitivity and slightly sensitivity.” The Road buffer zone (Fig 3h) forms distinct rings of medium-to-high sensitivity belts along expressways and arterial road corridors, demonstrating the cumulative effect of linear infrastructure in fragmenting and disturbing ecological patches. A notable example is Dianshan Lake to Yuandang Lake, which is segmented into two discontinuous parts by roads. Density of population (Fig 3i) and Nighttime light (Fig 3j) indicate that areas with high values—such as urban clusters (e.g., around Wujiang District government, Xujing Town government, the border of Qingpu and Minhang), industrial parks (e.g., the southern chemical industry zone in Wujiang District), and transportation hubs (e.g., Nanhui Station, Zhaoxiang Bus Station), which are mostly classified as slightly sensitivity and insensitivity. In contrast, the peripheral farmlands, woodlands, and wetland mosaic areas with low nighttime light and population density correspond largely to highly and extremely sensitivity levels. Furthermore, combined with the land use image, these three maps (Fig 3 f, i, j) show a high degree of spatial overlap in their insensitivity and slightly sensitivity areas, reinforcing the spatial contrast of “high development correlates with low ecological sensitivity, and weak development correlates with high ecological sensitivity.” This indicates that in plains with highly concentrated populations and economic activities, the remaining natural and farmland ecological patches are extremely sensitive to new disturbances and should be prioritized for protection.

In summary, geographical factors play only a secondary, background role in the ES of the YRD EGI-DDZ. Instead, water body proximity, land use and vegetation status, and development intensity indicators (population density and nighttime light) jointly determine the primary spatial pattern of sensitivity. High sensitivity zones, centered on river or lake shorelines and wetlands, along with peripheral woodlands, grasslands, and some farmland with low development intensity, constitute the main body of high sensitivity areas. Urban and high-intensity construction zones primarily fall within low-sensitivity and insensitive categories. This result clearly demonstrates a shift in the controlling mechanism of ecological sensitivity within the plain river network-urban composite system: from geography-controlled to land use, human disturbance, and hydrology combined control.

### 3.2. Results of AHP

The geometric mean of their ratings was calculated to construct the judgment matrices, which were then subjected to standard consistency tests. The article presents a detailed process (Table 7–10) for evaluating criterion levels, and the same method is applied to each indicator level. Finally, the proportions of each factor were calculated and integrated (Table 11).

**Table 7:** AHP Data.

AHP Data				
	Geological Sensitivity	Hydrological Sensitivity	Natural Resource Sensitivity	Human Interference Sensitivity
Geological Sensitivity	1.000	0.149	0.133	0.141
Hydrological Sensitivity	6.700	1.000	0.385	0.526
Natural Resource	7.500	2.600	1.000	1.449
Human Interference Sensitivity	7.100	1.900	0.690	1.000

**Table 8:** AHP Results.

AHP Results				
**Factors**	**Eigenvector**	**weight**	$\lambda_{max}$	**CI**
Geological Sensitivity	0.177	4.4211%	4.074	0.025
Hydrological Sensitivity	0.821	20.5136%		
Natural Resource Sensitivity	1.715	42.8628%		
Human Interference Sensitivity	1.288	32.2025%		

**Table 9:** Random consistency RI table.

Random consistency RI table														
**n**	3	4	5	6	7	8	9	10	11	12	13	14	15	16
**RI**	0.52	0.89	1.12	1.26	1.36	1.41	1.46	1.49	1.52	1.54	1.56	1.58	1.59	1.59
**n**	17	18	19	20	21	22	23	24	25	26	27	28	29	30
**RI**	1.61	1.61	1.62	1.63	1.64	1.64	1.65	1.65	1.66	1.66	1.66	1.67	1.67	1.67

**Table 10:** Summary of consistency test results.

Summary of consistency test results				
$\lambda_{max}$	**CI**	**RI**	**CR**	**Consistency check results**
4.074	0.025	0.890	0.028	PASS

**Table 11:** The final result of the proportions of each factor.

Objective Layer	Criterion Layer	Weight of Indicator	Indicator Layer	Weight of Criterion	Final Weight
Ecological Sensitivity	Geological Sensitivity	4.42%	Elevation/m	64.52%	2.85%
			Slope/°	35.48%	1.57%
	Hydrological Sensitivity	20.51%	River buffer zone/m	69.93%	14.34%
			Lake buffer zone/km	30.07%	6.17%
	Natural Resource Sensitivity	42.86%	Surface type	19.05%	8.16%
			Land use	48.09%	20.63%
			Fractional Vegetation Cover	32.86%	14.08%
	Human Interference Sensitivity	32.2%	Road buffer zone/m	20.41%	6.57%
			Density of population	34.64%	11.15%
			Night lighting	44.96%	14.48%

The random consistency index (RI) for a 4th-order matrix is 0.890, as established by standard RI tables. This value is used in the subsequent consistency ratio calculation.

The AHP results reveal a clear hierarchy among the four criteria: Natural Resource Sensitivity holds the highest importance (42.86%), followed by Human Interference Sensitivity (32.20%) and Hydrological Sensitivity (20.51%), while Geological Sensitivity plays only a minor role (4.42%). This reflects that, in the low-relief plain of the YRD EGI-DDZ, ES is primarily shaped by resource conditions and human disturbance rather than by geographical variation. At the indicator level, land use, nighttime lights, river buffer zone, and FVC emerge as the most influential factors, collectively accounting for the majority of the total weight. This weighting structure is consistent with the characteristics of a highly urbanized plain river-lake system, where anthropogenic activities and landscape composition exert dominant control over ecological sensitivity, while topographical factors contribute marginally.

### 3.3. Comprehensive ES Analysis of YRD EGI-DDZ

**Fig 4:**
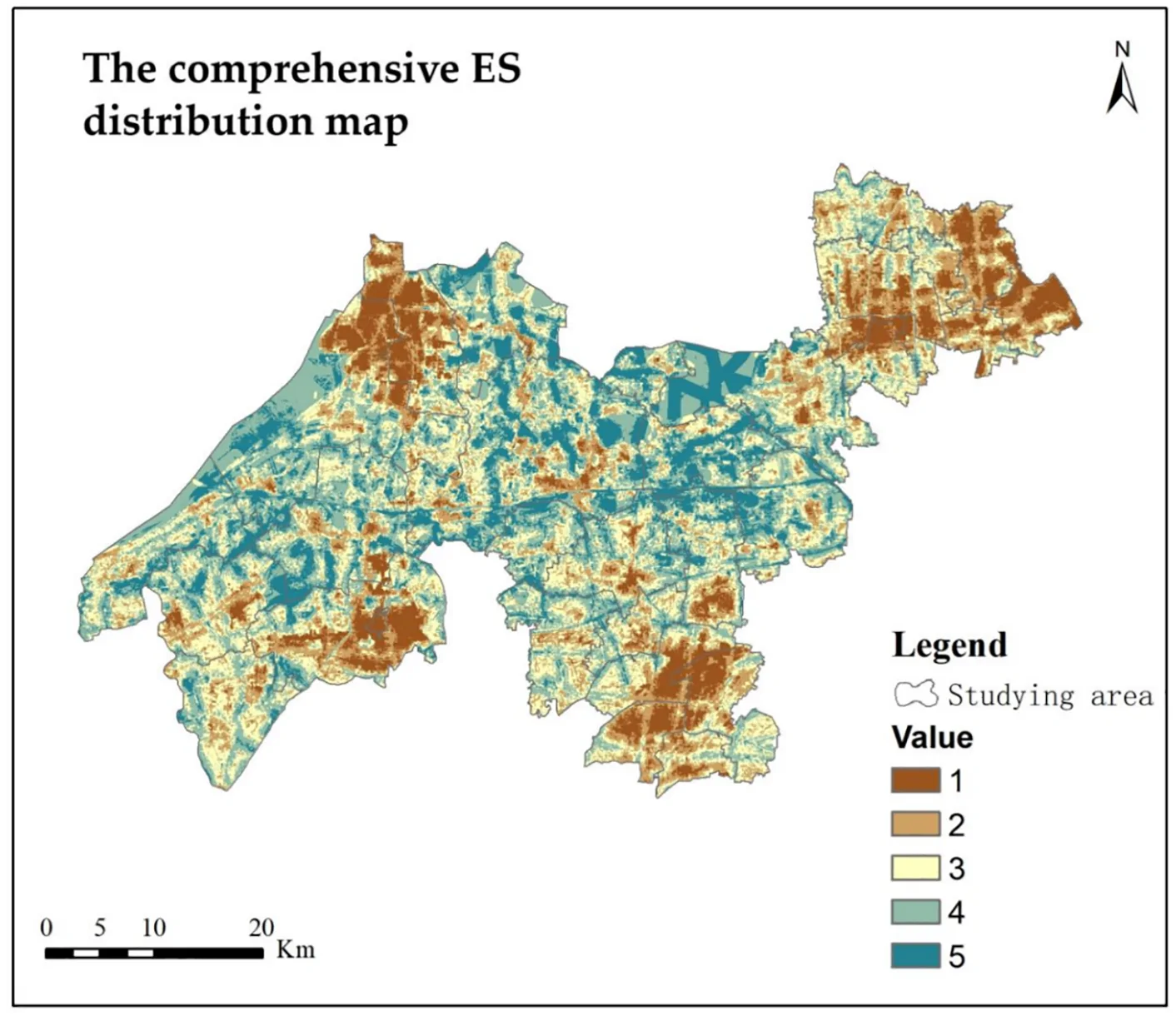
The comprehensive ES distribution map of YRD EGI-DDZ. Base map data obtained from the China Standard Map Service, Ministry of Natural Resources of the People’s Republic of China (http://bzdt.ch.mnr.gov.cn/). Map approval number: GS (2020) 3189.

Base on the AHP weighted overlay, the composite ES map (Fig 4) highlights several distinctive features of the YRD EGI-DDZ. In area terms (Table 12), extremely and highly sensitive zones (classes 5 and 4) together account for 43.26% of the study area, moderately sensitive zones (class 3) for 26.85%, while slightly sensitive and insensitive zones (classes 2 and 1) sum to 29.89%. Spatially, extremely and highly sensitivity areas are mainly associated with contiguous forest and high-coverage grassland patches, and the shorelines and water and land transition zones of major rivers, lakes, tidal flats and marshes. Slightly sensitivity and insensitive zones are largely concentrated in urban land, rural settlements and other built-up areas, as well as intensively used farmland. However, the composite ES map also shows that these classes are finely interwoven: highly sensitivity patches are frequently embedded within or cut by low sensitivity built-up areas and transport corridors, resulting in a pronounced “mosaic” and fragmented ecological pattern. Dense expressway and road networks intersecting with river and lake systems lead to a visible “cut-up” of otherwise continuous high-sensitivity belts, with the Dianshan Lake to Yuandang Lake shoreline and adjacent wetlands being a particularly striking example.

**Table 12:** Comprehensive evalution analysis of ES.

Ecological sensitivity evaluation grade table	Comprehensive evaluation index	Acreage/ ha	Proportion %
Extremely sensitivity	3.89-4.88	35066.10	14.59%
Highly sensitivity	3.50-3.89	68906.46	28.67%
Moderately sensitivity	3.09-3.50	64532.20	26.85%
Slightly sensitivity	2.61-3.09	45400.87	18.89%
Insensitivity	1.40-2.61	26437.77	11.00%

At the sub-regional scale, the three constituent counties or districts exhibit differentiated sensitivity structures. In Qingpu District, low-sensitivity zones are concentrated in the northeast (around Xujing Town and Baihe Industrial Park) and extend southwestwards towards the Qingpu district government area. This belt coincides with a very dense transportation network, including multiple expressways (Shanghai Ring Expressway, Shenhai, Huyu, Huchang, etc.), elevated roads (e.g., Songze Elevated) and major arterial roads, which collectively reduce ecological sensitivity but also intensify the fragmentation of surrounding medium- and high-sensitivity patches. In Jiashan County, low-sensitivity areas are mainly clustered in the southern part of the site (around the county government) and scattered in the north. Unlike Qingpu, where road density is the main driver, these low sensitivity zones are dominated by residential, commercial and some industrial land; within them, fragmented medium and high sensitivity green spaces and scenic spots are embedded as isolated ecological patches. In Wujiang District, parts of East Taihu Lake and the dense internal network of lakes and rivers result in an overall pattern where low-sensitivity zones are located in the north (around the district government and several urban transport hubs and bus or coach stations) and in the south (industrial parks), while the central and western parts form a relatively continuous band of high sensitivity associated with East Taihu and connected water and land transition zones. This “low sensitivity ring surrounding a high-sensitivity core” further underscores the tension between intensive urban and industrial development and the protection of key aquatic and wetland ecosystems in this highly urbanized plain river and lake system.

In summary, in addition to the overall dominance of medium to high sensitivity classes, the composite ES map also reveals a pronounced spatial fragmentation of sensitivity patterns across the YRD EGI-DDZ. High and extremely sensitive patches are interspersed with low-sensitivity and built-up areas at fine scales, especially around urban fringes and along major transport corridors, forming a typical “mosaic” of ecological and development spaces. This indicates that the remaining ecologically sensitive land in the plain is highly fragmented and frequently intersected by construction land, which not only increases edge effects and disturbance risks, but also poses challenges for maintaining continuous ecological corridors and for implementing integrated, cross-boundary conservation measures.

### 3.4. Driving force analysis of ES

**Fig 5:**
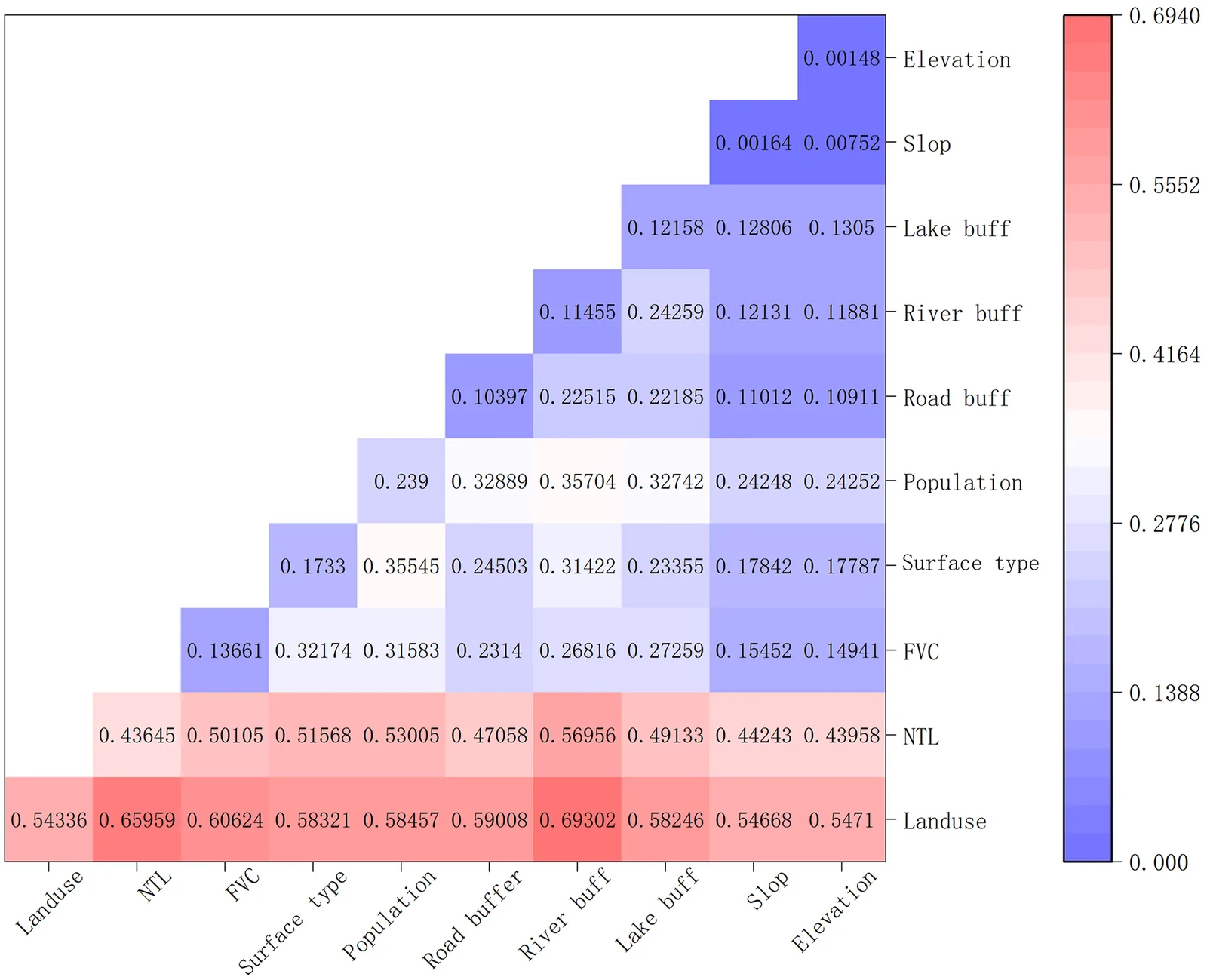
Multi-factor interaction-driven results for ES.

The q-values (Fig 5, Table 13) reveal a clear hierarchy of explanatory power among the selected factors. Land use (q = 0.543, p < 0.001) emerged as the dominant driver, followed closely by nighttime light intensity (q = 0.436, p < 0.001), both falling within the strong explanatory category (q ≥ 0.30). Population density (q = 0.239, p < 0.001) demonstrated moderate explanatory power (0.10 ≤ q < 0.30), together with the natural and infrastructure-related factors—surface type (q = 0.173), FVC (q = 0.137), lake buffer (q = 0.122), river buffer (q = 0.115), and road buffer (q = 0.104), all of which were statistically significant (p < 0.001). In contrast, elevation (q = 0.001) and slope (q = 0.002) yielded negligible q‑values (q < 0.10), confirming their marginal role in this low‑relief plain setting. This result clearly indicates that, in the low-relief plain of the YRD EGI-DDZ, geographical variation is negligible as a driver of ES spatial differentiation, in sharp contrast to mountain and plateau regions where elevation and slope usually play a dominant role.

**Table 13:** The q-statistics on factors inﬂuencing ES.

	Land use	NTL	FVC	Soil type	Popul ation	Road buff	River buff	Lake buff	Slop	Elev ation
**q**	0.54336	0.43645	0.13661	0.17330	0.23900	0.10397	0.11455	0.12158	0.00164	0.00148
**p**	0.000	0.000	0.000	0.000	0.000	0.000	0.000	0.000	0.88984	0.98515

Overall, the Geodetector results corroborate the AHP based ranking in highlighting land use and human disturbance as key controls, while further quantifying their relative importance and revealing the marginal role of geography in this highly urbanized plain river and lake system.

### 3.5. Land use dynamics and ES changes from 2018 to 2020

#### 3.5.1. Land use transition patterns and sensitivity structure changes.

**Fig 6:**
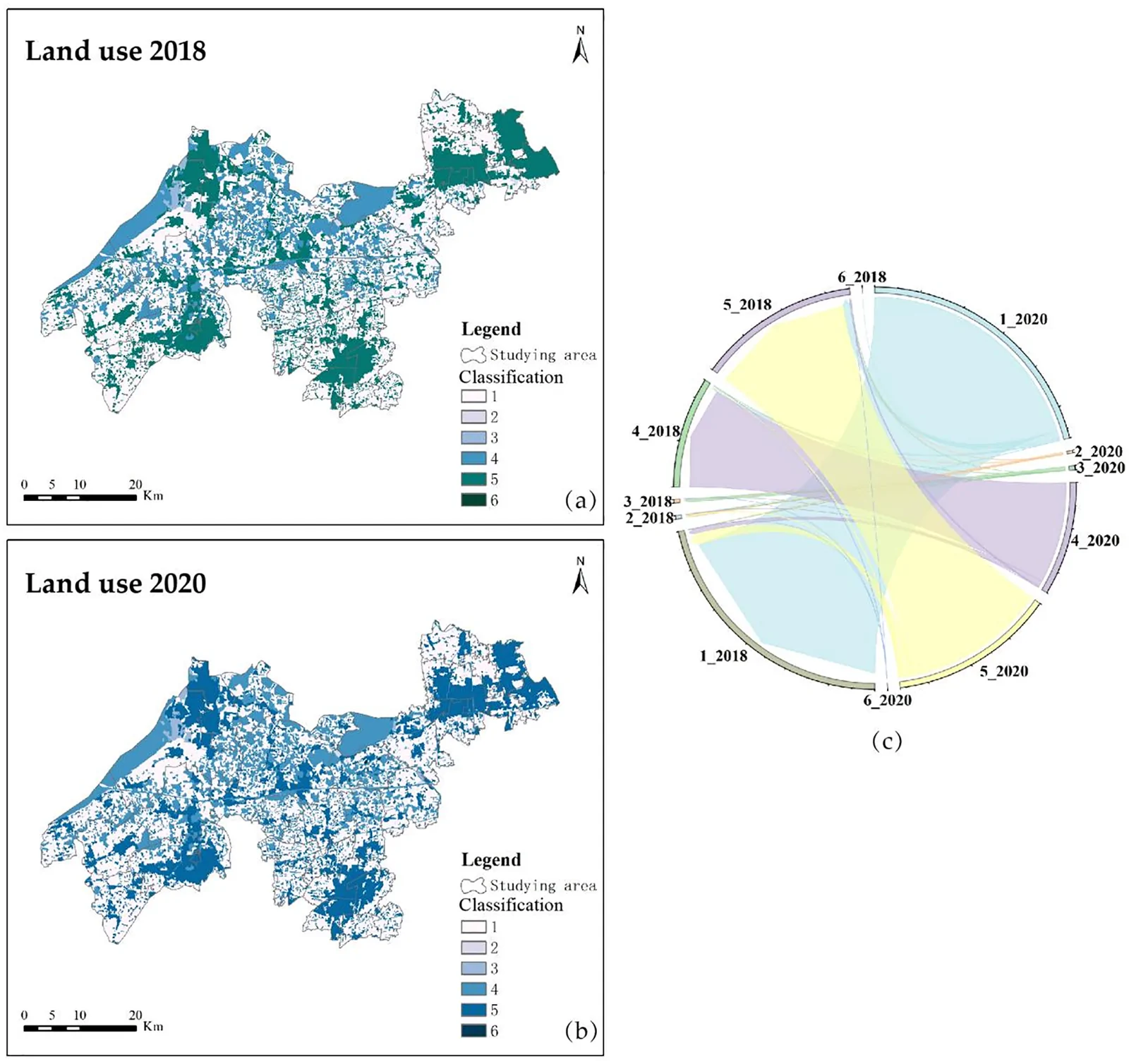
LUC from 2018 to 2020. a, Land use 2018; b, Land use 2020; c, Land use transition chord diagram 2018-2020. Base map data obtained from the China Standard Map Service, Ministry of Natural Resources of the People’s Republic of China (http://bzdt.ch.mnr.gov.cn/). Map approval number: GS (2020) 3189.

From 2018 to 2020, LUC in the YRD EGI-DDZ was characterized by overall stability of major categories, superimposed with a clear trend of cropland being converted to construction land (Fig 6, Table 14). The transition matrix based on six functional classes shows that cropland, water bodies and construction land remained largely unchanged along the diagonal, with 1,096.40 km² of cropland, 467.62 km² of water and 665.12 km² of construction land persisting between 2018 and 2020. The most prominent conversion was from cropland to construction land, with about 123 km² transferred from class 1 to class 5, indicating that recent urban expansion has mainly occurred at the expense of agricultural land rather than large-scale direct occupation of ecological land or water bodies.

**Table 14:** The land use transition matrix.

2018		2020					
		Cropland	Forest	Grassland	Water Bodies	Artificial Surfaces	Unused Land
		1	2	3	4	5	6
**Cropland**	1	1096.4007	2.5947	1.2258	23.4108	50.5791	0.1449
**Forest**	2	1.8369	7.8381	1.944	1.1223	1.5084	--
**Grassland**	3	0.144	0.0009	15.4962	0.2079	1.5498	0.009
**Water Bodies**	4	11.2617	0.4806	0.3582	467.6193	5.9616	0.8721
**Artificial Surfaces**	5	34.0281	0.8235	0.1674	6.7014	665.1216	0.0036
**Unused Land**	6	0.0018	--	--	--	--	--

The land use sensitivity structure also changed slightly over the same period (Table 15). The proportion of insensitive land decreased from 18.90% in 2018 to 14.69% in 2020, while slightly sensitive land increased from 11.23% to 16.17%, and highly and extremely sensitive land (classes 4–5) together rose from 21.04% to 21.72%. Moderately sensitive land (class 3) remained the dominant category, declining only marginally from 48.83% to 47.42%. These changes suggest that, although construction land has expanded into medium sensitivity cropland belts, some internal greening and the relative stability (or slight increase) of highly sensitive water and ecological patches have led to a shift in the overall structure towards a higher share of medium-to-high sensitivity classes. Combined with the dense road and river and lake networks, this process helps explain why the current ES pattern exhibits both generally high sensitivity and pronounced spatial fragmentation, with high sensitivity cores increasingly surrounded and dissected by low-sensitivity development zones.

**Table 15:** The percentage of Land use areas-based sensitivity levels 2018 vs 2020.

The percentage of Land use areas-based sensitivity levels 2018 vs 2020					
Period	In- sensitivity	Slightly sensitivity	Moderately sensitivity	Highly sensitivity	Extremely sensitivity
2018	18.90%	11.23%	48.83%	0.79%	20.25%
2020	14.69%	16.17%	47.42%	0.97%	20.75%

#### 3.5.2. Implications of land use for the fragmentation of ES and sustainable planning.

**Fig 7:**
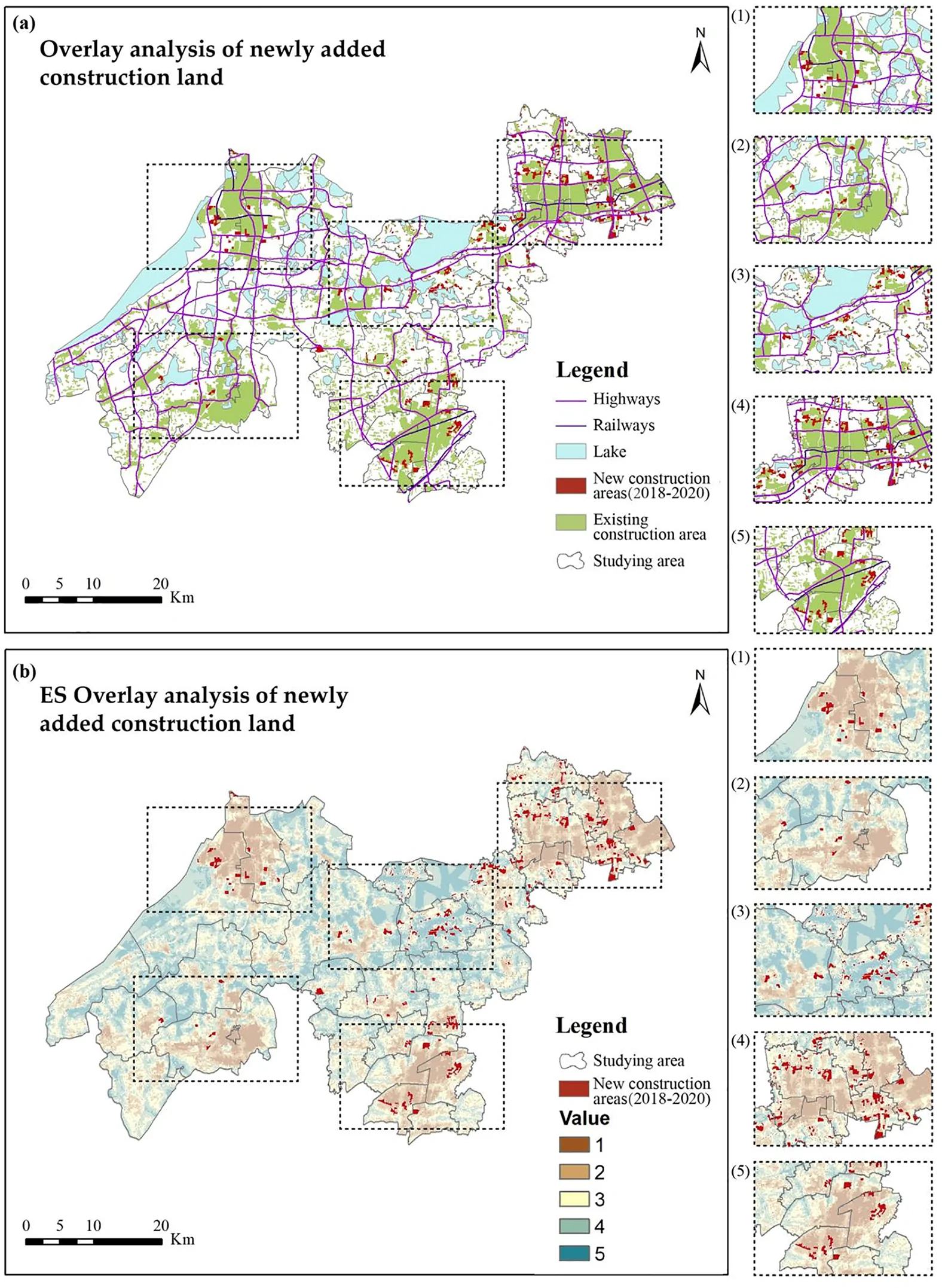
Overlay analysis. a, Overlay analysis of newly added construction land; b, ES Overlay analysis of newly added construction land. Base map data obtained from the China Standard Map Service, Ministry of Natural Resources of the People’s Republic of China (http://bzdt.ch.mnr.gov.cn/). Map approval number: GS (2020) 3189.

Based on land use data from 2018 and 2020, and using ArcGIS’s raster calculator, data on newly added construction land from 2018 to 2020 were extracted. At the same time, the new construction land will be overlaid with roads, original construction land and comprehensive ES maps (Fig 7). Further clarifies where recent expansion has occurred within the sensitivity structure.

Overlay analysis reveals that the newly added construction land from 2018 to 2020 was highly correlated with existing built-up areas and transportation corridors. The new patches generally exhibited the characteristics of expanding outward along the existing urban edges and developing in a linear pattern along expressways and major arterial roads. The most significant expansion occurred around the periphery of the urban area in northeastern Qingpu (Fig 7-a4), extending southwestward along major transportation corridors, which means that Qingpu District has the most prominent urbanization; Secondly, the newly built urban area is mainly located around the southern part of Jiashan County town and near the northern urban belt and southern industrial park areas in Wujiang. Most of the newly added construction areas were distributed adjacent to the 2018 existing built-up areas, forming continuous or semi-continuous expansion belts. Isolated “leapfrog” development far from towns and main roads was rarely observed. Simultaneously, the newly added construction land was distinctly aligned along expressways and rapid roads. Areas around expressway interchanges, key traffic nodes, and railway surroundings emerged as hotspots for new development. This development model, characterized by “reliance on the road network and expansion along cities,” on one hand, thickened the original low and slightly sensitive development belts. On the other hand, it spatially wedged into medium sensitivity and even locally high sensitivity areas, further fragmenting the originally more continuous ecological patches. This pattern reinforced the fragmented and “development corridors encircling high sensitivity cores” configuration evident in the 2020 ES map.

Furthermore, overlaying the newly added construction land with the 2020 ES map provides a clear visual confirmation: this “road-reliant, city-adjacent” expansion pattern intensified the original low and slightly sensitive development belts while progressively encroaching into medium and even high sensitivity zones. Consequently, the once relatively contiguous high and extremely high sensitivity patches were segmented into smaller fragments, accentuating the spatial fragmentation of the ES pattern. Particularly around the Dianshan Lake to Yuandang Lake shoreline and the corridor from northeastern to southwestern of Qingpu (Fig 7-b4), the new construction notably thickened the low sensitivity development ring, forming a more complete “encirclement” around the high sensitivity core areas. However, the ES assessment results of the north-south corridor along East Taihu Lake and the east-west corridor leading to Chenghu Lake are high and have not been significantly affected by the fragmentation of newly built urban areas, indicating that these routes have good ecological connectivity. Notably, the ecological connectivity of Dianshan Lake to Yuandang Lake had already been compromised by roads and existing construction. In its southern part (Fig 7-b3), the new development interlaces with a complex lake-river system, corresponding predominantly to medium and high sensitivity ES levels. This area should be designated as a key zone for stringent protection and restoration in subsequent planning and management.

In summary, these findings have direct implications for sustainable spatial planning in the YRD EGI-DDZ and can be aligned with current territorial spatial planning and the overall plan for ecological green integrated development. First, the extremely and highly sensitive zones (ES classes 4–5), which are concentrated along Dianshan Lake, Yuandang Lake, East Taihu, major rivers and associated wetlands and forest-grassland complexes, should be incorporated into strict protection zones and ecological source areas where new construction is prohibited and ecological restoration and corridor enhancement are prioritized. Second, the moderately sensitive belts at the urban fringe, where most new construction has occurred should be designated as key regulation zones, with explicit controls on additional linear expansion along highways and elevated roads, limits on construction intensity and impervious surface ratios, and requirements to maintain or create continuous blue and green buffers between development corridors and high sensitivity cores. Third, slightly sensitive and insensitive areas, mainly existing urban and industrial land, can serve as priority development and renewal zones under the condition that internal greening and blue and green infrastructure are strengthened to mitigate edge effects on adjacent medium and high sensitivity areas. In this way, the land use induced fragmentation patterns revealed in this study are translated into protection-regulation-development zoning and corridor control strategies, providing a sensitivity-oriented evidence base for more integrated and sustainable spatial planning across Qingpu, Jiashan and Wujiang.

## 4. Discussion

### 4.1. Comparison with previous researches

This study applies an integrated AHP-GIS, Geodetector, and LUC analysis to examine ES patterns in the YRD EGI-DDZ. The AHP-based ES assessment indicates a landscape dominated by medium to high sensitivity, with the most sensitive zones concentrated along major waterways, lakes, wetlands, and contiguous ecological land, contrasting with low sensitivity urban and industrial areas. Then, Geodetector results reveal that, in this flat plain, ES is chiefly determined by land use structure and human disturbance, supported by hydrological proximity and ecological base conditions, while geographical factors like elevation and slope show minimal influence. Final, the analysis of 2018–2020 LUC further shows that new construction primarily expands along existing urban edges and transport corridors. This pattern encroaches into medium sensitivity belts and increasingly surrounds high sensitivity cores, thereby exacerbating ES fragmentation.

In terms of the research findings, the main findings of this study are largely consistent with and effectively complementary to the recent research conclusions of scholars on YRD EGI-DDZ and the broader region. A prior InVEST model research, assessed habitat quality and pointed out that from 2000 to 2020, the rapid expansion of construction land at the expense of cultivated land and wetlands led to an overall decline in habitat quality [24]. The study found that areas of medium habitat quality were mainly associated with cropland and grassland, high quality habitats were concentrated in water bodies and wetlands, while poor-quality habitats were predominantly construction land. Our ES mapping and LUC analysis reached very similar conclusions: medium sensitivity zones are primarily cultivated land and general ecological land; High and extremely high sensitivity zones cluster along major lakes, rivers, and wetlands; and low sensitivity zones are largely confined to urban and industrial land. Furthermore, recent expansion of construction land has mainly resulted from the conversion of cultivated land belts.

Another coordination-focused study found that while overall territorial environmental coordination is balanced, ecological high value areas concentrate along the Taihu Lake corridor, and urban coordination depends on land use structure and policy rather than mere proximity to built-up land [26]. Our integrated AHP-GIS-Geodetector analysis supports this, showing ES patterns are driven by land use, human disturbance, and hydrological proximity—not simple urban distance or geography. It also reveals that high-sensitivity cores around Dianshan Lake and East Taihu are increasingly fragmented and encircled by road- and fringe-driven development corridors. From a network perspective, MSPA-based research highlights the fragmentation and pollution of the Yangtze River Delta region’s aquatic ecological matrix, urging a multifunctional blue-green infrastructure network to restore connectivity [42]. Our results on ES and LUC echo this fragmentation diagnosis. We advance this perspective by quantitatively identifying key drivers in the plain river and lake system and explicitly linking sensitivity patterns and LUC induced fragmentation to “conservation-regulation-development” zoning and corridor control strategies.

Methodologically, most existing research follows a general technical framework of “multi-criteria comprehensive evaluation + spatial statistics or detection + driving mechanism analysis.” For instance, Ren, Li et al. developed landscape risk or landscape ecological risk indices based on multi-period land use/cover (LUCC) data, and employed spatial autocorrelation, GeoDa/GWR, and Geodetector to reveal the spatiotemporal patterns of risk and identify dominant factors, such as temperature, GDP, and land use efficiency at provincial or watershed scales [43,44]. Xu combined the AHP with Geodetector for suitability assessment of development in ethnic minority villages, highlighting the integrated weighting of natural–social–cultural factors and the ranking of their q-values [45]. Overall, these studies collectively reflect a common technical pathway: first constructing an indicator system through AHP composite weighting, then applying methods such as Geodetector to identify dominant driving factors, ultimately supporting regional ecological conservation and sustainable development.

Compared to existing research, the methodology of this study demonstrates both continuity and innovation. The similarity lies in adopting the general framework of “multi-criteria comprehensive evaluation + spatial statistics/detection,” consistent with the work of Ren, Li, Xu et al. Specifically, it utilizes AHP-GIS to construct an indicator system and introduces Geodetector to quantitatively identify dominant driving factors. This continues the integrated approach of combining “subjective weighting (AHP/composite weighting) + data-driven diagnostics (Geodetector, GWR, etc.).” In comparison, this integrated approach, combining “weight determination” with “identification of dominant factors” through both expert judgment and objective data, and serves more directly in sensitivity zoning and the optimization of the indicator system. Furthermore, it closely links the conclusion that land use plays a dominant role in ecological sensitivity on plains with spatial planning. Through analysis of 2018–2020 land use dynamics and the overlay of newly added construction land on the ES map, the study quantitatively reveals how expansion along urban edges and transportation networks wedges into medium sensitivity belts, fragments high sensitivity patches, and thickens low sensitivity development corridors.

In summary, the study proposes sensitivity oriented “conservation-regulation-development” zoning and corridor control recommendations tailored to the demonstration zone, directly aligning with policy instruments such as national and regional ecological environmental special plans and integrated action plans. Within the integrated chain of “assessment-driving mechanism-planning”, this work thus deepens the decision support role of Geodetector in ecological risk and vulnerability studies and provides a sensitivity and planning oriented complement to existing analyses of habitat quality evolution, coordination assessment and landscape infrastructure network design. Together, these contributions reinforce a common consensus: spatial planning in the Ecological Green Integrated Development Demonstration Zone must give high priority to the impacts of land use and human disturbance and commit to a management framework based on robust blue and green ecological corridors.

### 4.2. Urban fragmentation in plain regions: Mechanisms and planning implications

Landscape ecology and ecological security pattern (ESP) theory provide a useful lens for interpreting our ES and LUC results in the YRD EGI-DDZ. ESP studies emphasize that the spatial configuration of ecological patches and corridors directly affects ecological processes such as species migration, material cycling and energy flow, and that a stable ESP is typically constructed through the “source identification-resistance surface-corridor extraction” paradigm based on MSPA, MCR and circuit theory [46–48]. In this framework, high-value habitats (ecological sources), low-resistance corridors and their connectivity determine the robustness and resilience of regional ecosystems. Our findings that high and extremely sensitive areas are concentrated along lakes, rivers and contiguous ecological land. However, these areas are increasingly fragmented and encircled by transport and fringe-oriented development corridors, which fully consistent with the concern in ESP theory about habitat fragmentation, broken corridors and “pinch points”. Although this study does not explicitly construct an ESP using MSPA or MCR, the AHP-GIS-Geodetector-LUC framework effectively identifies high sensitivity “source-like” areas and low sensitivity “development corridors”, and reveals where current land use dynamics are undermining potential ecological connectivity in this plain river and lake system. In this sense, our sensitivity-oriented protection-regulation-development zoning and corridor control suggestions can be seen as a complementary. The results provide a practical bridge between ESP theory and the ongoing, policy driven efforts to improve ecological connectivity and resilience in the YRD demonstration zone.

As a further validation of this study’s practical relevance, a comparison was conducted between our results and the document “Special Ecological and Environmental Plan for the YRD EGI-DDZ (2021-2035) “ issued by the Shanghai Municipal Bureau of Ecology and Environment in 2023. A “One Core, Two Corridors, Three Chains, Four Zones” overall spatial framework has been proposed for the area. The Plan states that by 2035, a natural ecological pattern characterized by “water as the vein, forest-field symbiosis, and city-green integration” should be fundamentally established. It aims to create a clean and safe aquatic ecosystem and a Jiangnan water townscape pattern integrating “points, lines, areas, and substrates”, thereby achieving coordinated symbiosis between ecology and development.

However, findings from this study indicate that while high sensitivity core areas such as Dianshan Lake and Yuandang Lake are designated as the “Ecological Green Core” and key corridor nodes within the overall layout, development belts along urban fringes and highways or arterial roads still noticeably wedge into medium sensitivity zones, fragment high sensitivity patches, and thicken low sensitivity development corridors. This results in a local-scale situation where the blue and green network, intended to be “water-veined,” appears encircled and fragmented.

**Fig 8:**
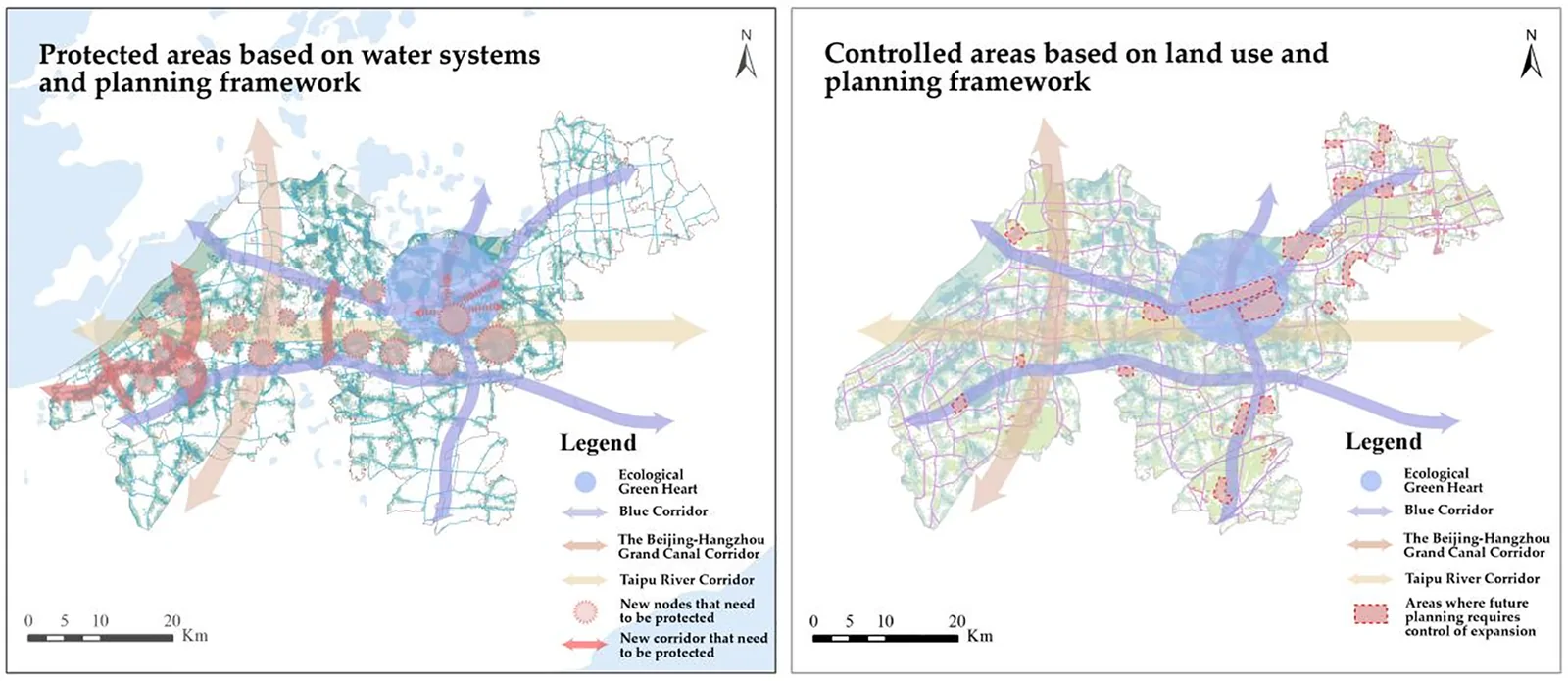
Sustainable Spatial Planning. Base map data obtained from the China Standard Map Service, Ministry of Natural Resources of the People’s Republic of China (http://bzdt.ch.mnr.gov.cn/). Map approval number: GS (2020) 3189.

The existing Plan predominantly sets goals and measures from functional and engineering perspectives, such as water quality compliance, sewage collection, and pollution source removal. It lacks sensitivity pattern-based, differentiated controls regarding the spatial relationships, development intensity, and corridor widths among high, medium, and low sensitivity development corridors. Building upon the Plan’s overall “One Core, Two Corridors, Three Chains, Four Zones” framework and its emphasis on the aquatic system alongside the site’s inherently fragmented landscape pattern, this study employed AHP-GIS to identify high and extremely high sensitivity zones. These were then overlaid with the site’s water system, newly added construction land (2018–2020), and the complex road network. This process clarified the ecological protection areas and development zones requiring controlled expansion within and around the “One Core” and “Two Corridors” (Fig 8).

The Figure illustrates that the proposed planning recommendations focus on river and lake shoreline restoration, wetland rehabilitation, and the establishment of blue and green buffer zones to prevent further intrusion by transportation and construction corridors into high sensitivity areas. Consequently, the sensitivity zoning can be integrated with existing objectives for the “One River, Three Lakes” water quality, the delineation of aquatic ecological conservation areas, and wetland protection inventories. This provides a refined, ecology sensitivity based spatial constraint and optimization layer for the governance pathway of “reduction, capacity increase, and quality improvement” and the integrated spatial framework. It aims to facilitate a transition in the demonstration zone’s development from merely functional compliance to a higher standard that achieves dual synergy between spatial pattern and ecological process.

### 4.3. Limitations and future research

Several limitations of this work merit discussion. First, due to limitations in data updates, this assessment is essentially a single baseline analysis centered around 2020. Although 2018–2020 LUC was used to diagnose recent development dynamics, the time window is relatively short, and longer multi-temporal datasets would be needed to fully reconstruct the evolution of ES patterns and fragmentation. Second, some driving factors, such as population density and nighttime lights, are only available for one or few years and were assumed to be temporally stable when approximating earlier conditions, which may underestimate temporal variation in human disturbance. Furthermore, we acknowledge that the VIIRS nighttime light data, originally at 500 m resolution, were resampled to 30 m using the Nearest Neighbor method to maintain spatial consistency with other datasets. While this rescaling does not enhance the effective resolution, the NTL data were used as a relative indicator of human activity intensity and subsequently classified into ordinal sensitivity grades (1–5), which reduces the sensitivity to absolute brightness values. Nevertheless, we recognize that the availability of higher resolution NTL data would improve the accuracy of future assessments. Third, the ES model is based on a linear AHP-GIS weighted overlay and does not explicitly incorporate non-linear interactions or feedbacks between factors, even though Geodetector provides some insight into factor importance. Similarly, the LUC analysis is descriptive and rule-based rather than a formal scenario simulation. Finally, ecological processes such as biodiversity, species movement and functional connectivity are captured indirectly through sensitivity patterns, but not explicitly modelled with dedicated landscape metrics or graph-based approaches.

In summary, future research could extend this framework by integrating longer term, higher resolution LUC and ecological data. For example, employing methods like AHP combined with entropy weighting or machine learning weighting to validate the robustness of factor weights. Then, introducing landscape connectivity and graph theory metrics to quantify fragmentation and the step effect, and developing simplified scenario-based land use simulations to assess the impacts of different planning schemes and corridor control strategies on ecological sensitivity and connectivity in highly urbanized plains.

## 5. Conclusions

This study developed an AHP-GIS-Geodetector framework to assess ES in the YRD EGI-DDZ, a highly urbanized plain river and lake system, and to link the results with recent LUC and sustainable spatial planning. Ten indicators were integrated into three dimensions: geological, hydrological ecological base and human disturbance and weighted by AHP, and a composite ES index was mapped. Geodetector was then used to quantify the contribution of each factor to the spatial differentiation of ES, and 2018–2020 LUC was analysed to interpret the observed pattern of sensitivity fragmentation and its relationship to urban expansion and road development.

The results show that the demonstration zone as a whole is in a highly sensitive ecological state, with more than 70% of the area falling into medium to extremely high ES classes, and highly and extremely sensitive zones clustered along Dianshan Lake, Yuandang Lake, East Taihu, major rivers, wetlands and contiguous ecological land. Low sensitivity areas are mainly existing urban and industrial land. Geodetector analysis reveals that, in this low-relief plain, ES is dominated by land use structure and human disturbance (land use, nighttime lights, population density), together with hydrological proximity and ecological base conditions (surface type and FVC), whereas elevation and slope have almost no explanatory power. This represents a clear shift from the geography-controlled sensitivity typical of mountainous and plateau regions to a land use and disturbance driven regime in a highly urbanized plain river and lake system.

The 2018–2020 land use analysis indicates that major land use categories are generally stable, with net expansion of construction land occurring mainly at the expense of cropland, while large water bodies and most ecological land remain relatively unchanged. However, new construction land is highly concentrated along existing urban fringes and expressways or arterial roads, particularly along the northeast to southwest development axis in Qingpu, around the county seat of Jiashan and near the northern urban belt and southern industrial parks in Wujiang. When overlaid with the ES map, these expansions are found primarily in slightly and moderately sensitive belts, carving low sensitivity development corridors into medium sensitivity zones and further fragmenting high and extremely high sensitivity patches, especially around Dianshan Lake and East Taihu. As a result, high sensitivity cores are increasingly encircled and dissected by development strips, reinforcing the mosaic and fragmented ES pattern.

These findings have direct implications for sensitivity oriented sustainable spatial planning in the demonstration zone. The ES maps and land use diagnostics support a zoning scheme, key protection and controlled development zones, that can be superimposed on the existing “one core, two corridors, three chains and four zones” framework. Extremely and highly sensitive areas along major lakes, rivers and wetlands should be incorporated into strict protection and restoration zones, with strengthened control of shoreline development and priority for wetland restoration and water ecology projects. Moderately sensitive belts at the urban fringe and along transport corridors should be designated as key regulation zones, with limits on further linear sprawl, requirements for continuous blue and green buffers and careful adjustment of land use functions. Slightly sensitive and insensitive areas within existing urban and industrial clusters can serve as priority zones for accommodating incremental development and renewal, provided that internal greening, sponge-city measures and blue and green infrastructure are enhanced to mitigate edge effects on adjacent medium and high sensitivity cores.

In conclusion, the AHP-GIS-Geodetector-LUC framework provides a spatially explicit, sensitivity-oriented evidence base that helps operationalize the policy goal of “ecological priority and green integrated development” in the YRD EGI-DDZ and offers a transferable approach for ES assessment and planning in other highly urbanized plain regions.

## Supporting information

S1 File The Yangtze River Delta administrative boundary vector data used in this study (Figures 1, 3, 4, 6, 7, and 8) were obtained from the Ministry of Natural Resources of the People’s Republic of China standard map service (data page: http://bzdt.ch.mnr.gov.cn). Data were downloaded on: 2025-12-10; the map review number shown on the webpage is: GS (2020) 3189. The base map data were used only for spatial overlay, analysis, and visualization (e.g., study-area location map). Processing steps included coordinate reference conversion, clipping to the study area. Visualization adjustments were limited to boundary styling, fills, scale bar, north arrow, and legend; no substantive modification to the spatial geometry or administrative boundary attributes was performed. The maps are presented for academic research and manuscript publication; the study’s analytical results are original to the authors, with base boundaries used solely as reference/background.
(DOCX)
