# Supplementary material for: Ecological sensitivity and sustainable spatial planning in a highly urbanized plain: An AHP-GIS and geodetector approach in the Yangtze River Delta Plain
Source: PLoS One. 2026 Sep 8;21(9):e0357598. doi: 10.1371/journal.pone.0357598 (PMC13552756; doi:10.1371/journal.pone.0357598)
Supplement: Supplementary file 1 [file pone.0357598.s001.docx]

Explanation regarding map copyright

Source and metadata:

The Yangtze River Delta administrative boundary vector data used in this study (Figures 1, 3, 4, 6, 7, and 8) were obtained from the Ministry of Natural Resources of the People's Republic of China standard map service (data page: http://bzdt.ch.mnr.gov.cn). Data were downloaded on: 2025-12-10; the map review number shown on the webpage is: GS (2020) 3189. The screenshot shows the Ministry of Natural Resources' explanation of the scientific research situation and the proper use of standard maps. This statement was added to the supporting information of the article (Pages 29, lines 1036-1047).

Processing and purpose:

The base map data were used only for spatial overlay, analysis, and visualization (e.g., study-area location map). Processing steps included coordinate reference conversion, clipping to the study area. Visualization adjustments were limited to boundary styling, fills, scale bar, north arrow, and legend; no substantive modification to the spatial geometry or administrative boundary attributes was performed.

The maps are presented for academic research and manuscript publication; the study's analytical results are original to the authors, with base boundaries used solely as reference/background.

Copyright and compliance:

The Ministry’s website indicates that maps produced from such datasets and publicly released should follow national map review/approval procedures. The dataset page displays the map review number GS (2020) 3189, which can serve as evidence that the necessary review procedures have been followed.

Supporting Screenshot documents available:

Figure 9: The screenshot available from: https://gi.mnr.gov.cn/202508/t20250819_2897593.html, and contains the following (translation):

Our department's standard map service system provides standard maps to the public free of charge. The system includes 275 maps of China, 93 world maps, and 16 thematic maps. The public can browse and download them free of charge. When using standard maps directly, the map approval number must be noted; if standard maps are edited or modified, the map approval process must be repeated.

Figure 10: The screenshot available from: <http://bzdt.ch.mnr.gov.cn>, and contains the following (translation):

The standard map is compiled according to the standards for drawing national borders in China and other countries around the world. It can be used for news and publicity purposes, illustrations in books and newspapers, background images for advertising displays, and base maps for handicraft designs. It can also serve as a reference base map for compiling publicly available maps. The public can browse and download the standard map for free. When using the standard map directly, the map approval number must be noted.

Figure 11: Download Yangtze River Delta map, and Drawing Approval Number is GS(2020)3189.
